# Juniperus indica Bertol. extract synergized with cisplatin against melanoma cells via the suppression of AKT/mTOR and MAPK signaling and induction of cell apoptosis

**DOI:** 10.7150/ijms.49423

**Published:** 2021-01-01

**Authors:** Xiao-Fan Huang, Hong-Wei Gao, Shan-Chih Lee, Kai-Fu Chang, Li-Ting Tang, Nu-Man Tsai

**Affiliations:** 1 Institute of Medicine, Chung Shan Medical University, Taichung, 40201, Taiwan, ROC.; 2 Department of Medical Laboratory and Biotechnology, Chung Shan Medical University, Taichung, 40201, Taiwan, ROC.; 3 Department of Pathology, Tri-Service General Hospital, National Defense Medical Center, Taipei, 11490, Taiwan, ROC.; 4 Department of Medical Imaging and Radiological Sciences, Chung Shan Medical University, Taichung, 40201, Taiwan, ROC.; 5 Department of Medical Imaging, Chung Shan Medical University Hospital, Taichung, 40201, Taiwan, ROC.; 6 Clinical Laboratory, Chung Shan Medical University Hospital, Taichung, 40201, Taiwan, ROC.

**Keywords:** melanoma, *Juniperus indica* Bertol., synergism, cisplatin, anti-proliferation

## Abstract

*Juniperus indica* Bertol. is an herbal plant that belongs to the genus *Juniperus*, which is commonly used in traditional medicine to refresh the mind and for diuretic use. However, few studies have reported the function of *J. indica* Bertol. Hence, this study aimed to investigate the anti-tumor and synergistic potential of *J. indica* Bertol. extract (JIB extract) for melanoma cells. Our results indicated the anti-melanoma activity of JIB extract. JIB extract induced cell cycle arrest at the G_0_/G_1_ phase and decreased cyclin and cdk protein expressions. In addition, AKT/mTOR signaling and MAPK signaling were inhibited by JIB extract to suppress melanoma cell growth and proliferation. Additionally, JIB extract induced B16/F10 cell apoptosis via the caspase cascade. According to the JIB extract's anti-melanoma capacity, to assess the synergistic effects of cisplatin and JIB extract. The results demonstrated that JIB extract combined with cisplatin enhanced the inhibition of cell growth, proliferation, and survival through the obstruction of cell cycle progression and AKT/mTOR and MAPK signaling as well as the induction of cell apoptosis. Collectively, our results indicate that JIB extract showed anti-tumor effects and synergized with cisplatin against B16/F10 cells, indicating the possibility of JIB extract to be developed as adjuvant therapy for melanoma.

## Introduction

Melanoma is the most malignant and lethal skin tumor owing to its high metastatic ability and the incidence of melanoma has been increasing over the years. The survival rate of melanoma patients with increasing stage ranges from 85% to 25% due to the high incidence of metastasis 1. The current treatments for melanoma are surgery, which is performed in the early stages of the disease, and neoadjuvant treatments for advanced patients, including chemotherapy, radiotherapy, targeted therapy, immunotherapy and combination therapy 2, 3. Among these, targeted therapy and immunotherapy are now the current main therapeutic procedure in melanoma. Targeted therapy drugs are used to target specific genes and proteins of cancer cells to precisely identify and attack specific types of cancer cells. Some studies revealed the rate of BRAF mutation is about 15-25% in Asian, which is significantly lower than that in Caucasians (50%-70%) 4. For instance, vemurafenib is a targeted therapy for melanoma with BRAF gene mutation. It can reduce the disease progression rate by 74% and improves outcomes for patients; however, about half of the patients will relapse again after five to six months 5. Besides, 12% of patients have second- or third-degree skin reactions that are sensitive to light, and about 18% of patients develop cutaneous squamous-cell carcinoma and keratoacanthoma or occurred at the same time 6. Immunotherapy helps to activate the immune system to fight cancer. For example, nivolumab is a monoclonal antibody and immune checkpoint inhibitors. It can target PD-1 in T cells to help T cells to recognize cancer cells and kill them through an immune response. The response rate is 32%, and the therapeutic effect only sustains six months. It can induce a severe immune reaction, hypofunction of thyroid glands, adrenal insufficiency, nephritis, and increment of the liver index 7. Ipilimumab is an immune checkpoint inhibitor that can inhibit CTLA-4 on human T cells to activate the immune system and attack cancer cells to achieve the effect of treating cancer. The response rate to melanoma was only about 10%, and only 20% of patients can achieve long-term survival 8. Pembrolizumab immune checkpoint inhibitor against PD-1. The response rate is 30%, which is better than ipilimumab. The side effects of pembrolizumab are similar to ipilimumab, including skin rash, diarrhea, abnormal liver function, and hypofunction of endocrine system function 9. Nivolumab, ipilimumab, and pembrolizumab are immune checkpoint inhibitors and can cause immune-mediated pneumonitis, colitis, hepatitis, and endocrinopathies nephritis, skin adverse reactions, encephalitis.

With recent advances in immunotherapy and targeted therapy which has improved the median OS for advanced melanoma, however, chemotherapy is also the backbone of systemic treatment for advanced melanoma for many years 10. Besides, in Taiwan, the use of target and immunotherapy is still very restricted. The first reason is that the most common melanoma in Taiwan is acral lentiginous melanoma, which is different from Western countries. The second reason is that less than 20% of patients in Taiwan have BRAF mutations, and patients who received targeted therapy will gradually develop resistance after one year. Therefore, most patients cannot benefit from targeted drug treatment. Moreover, although immunotherapy is not limited to specific gene mutations, its expensive medicines are not affordable by the general public. For the above reasons, most of the treatments for metastatic melanoma in Taiwan are still based on traditional combined chemotherapy. For example, platinum-based chemotherapy drugs, such as cisplatin, carboplatin, paclitaxel, or dacarbazine (DITC). Cisplatin is used as a chemotherapeutic agent for the treatment of several cancers and is commonly applied for neoadjuvant or adjuvant therapy at different stages of cancer therapy. In addition, many clinical trials have used cisplatin in the treatment of melanoma 11, 12. Cisplatin- paclitaxel -dacarbazine regimen has shown that the response rate of 41% and the median overall survival time is 11 months 13. Carboplatin plus paclitaxel has revealed that the median progression-free survival is 4.2 to 4.4 months, and the median overall survival time 9 to 11 months 14. These chemo-drugs may also cause nephrontoxicity, ototoxicity, neurotoxicity, cardiotoxicity, hepatotoxicity, hematological toxicities, and gastrointestinal toxicity. Hence, these anticancer agents provide good survival benefits for patients and cause different side effects during the therapeutic procedure. It is hoped to find new combined chemotherapy drugs with low physiological toxicity to reduce side effects and overcome the current limitations of use and be widely used clinically, thereby bringing patients' most tremendous benefits.

The natural product is the primary source of the development of drugs, and several current compounds have been used as a standard therapeutic drug on the various treatment of disease. Moreover, natural extracts exert several bio-functions all in one, such as anticancer, anti-inflammatory, antioxidative activity. Besides, natural extracts not only have been reported its' anticancer activity in several types of plants but also demonstrated its' effects on reducing the side effects in the therapeutic procedure. For instance, *Bacopa monnieri* extract may be useful alone or in combination with other anti-emetic drugs to treat cisplatin-induced emesis in man 15. Nigella sativa oil ameliorates the effect of cisplatin-induced gastrointestinal dysfunction 16. *Juniperus indica* Bertol. is an evergreen tree that is distributed in high-altitude regions with low temperatures 17, 18. A few scientific studies have reported on the use of *J. indica* Bertol. It has been found to have anti-microbial and cytotoxic activities 19. Nevertheless, *J. communis* which is classified to *Juniperus* genus and is the same genus but different species as *J. indica* Bertol. have fully investigated several bio-functions. For example, *J. communis* exhibits hepatoprotective potential against paracetamol and azithromycin induced liver injury in rats 20, ameliorates tacrolimus-induced nephrotoxicity in rats 21, exerts anti-hypoglycemic and anti-hypolipidemic effects in alloxan-induced diabetic rats 22, and treats the neurasthenic neurosis, and against Freund's adjuvant-induced arthritis in rats 23. However, the bio-functions of *J. indica* Bertol. (JIB extract) have not been fully explored. Here, we aimed to investigate the synergistic effects of JIB extract combined with cisplatin on melanoma to reduce the cisplatin concentration to prevent the unpredictive clinical outcome.

In our study, JIB extract significantly inhibited B16/F10 cell growth and impeded cell cycle at G_0_/G_1_ phase and triggered cell death through apoptosis. Furthermore, JIB extract combined with cisplatin has synergistic effects on the enhancement of cell repression, induction of cell apoptosis, interruption of AKT/mTOR signaling, and MAPK signaling. Consequently, JIB extract can be potentially utilized as an anti-melanoma agent and adjuvant treatment in the future and this study might provide a new therapeutic strategy for patients who do not have BRAF mutations and cannot receive immunotherapy.

## Materials and methods

### Cell culture

The B16/F10 (Mouse skin melanoma) and MDCK (Canine kidney epithelial cell) cell lines were purchased from the Bioresource Collection and Research Center (Hsinchu, Taiwan). MDCK cell line was used as an example for kidney toxicity assay in this study. Both cell lines were cultured in DMEM medium supplemented with 10% FBS, 1% sodium pyruvate, 1% HEPES, and 1% penicillin/streptomycin in a humidified incubator with 5% CO_2_. Cells were sub-cultured with 0.05% trypsin-EDTA for the follow-up experiment. All cell culture reagents were purchased from Gibco (Grand Island, NY, USA).

### Preparation of *Juniperus indica* Bertol. extract (JIB extract)

A fresh *Juniperus indica* Bertol. plant from Nepal was utilized, and the extract was obtained by steam distillation. The small-scale extraction was done in our lab and the detailed conditions were described as the following. The fresh fruits of *Juniperus indica* Bertol. (400g) were placed in a 2-L steam distillation steel apparatus unit and the generated steam passed through plant material for 100 mins at 100~105℃ and a flow rate of approximately 7.2 ml/min. The large scale of JIB extract was commissioned by Phoenix (New Jersey, USA). The JIB extract was preserved in an airtight and lightproof aluminum can and stored at 4°C. Before conducting the experiments, the JIB extract was dissolved in DMSO and measured in μg/ml. The concentration of JIB extract = the weight of 20 μl JIB extract (g)/ (the weight of 180 μl DMSO + the weight of 20 μl JIB extract) (g).

### Cell viability of cells

Cell viability was assessed by MTT assay. B16/F10 (5 × 10^3^) and MDCK (1 × 10^4^) cells were grown in 96-well plates and treated with JIB extract (0-100 μg/ml), cisplatin (0-10 μg/ml), and 5-FU (0-2 μg/ml) for 24, 48, and 72 hrs, respectively. After incubating the cells with these drugs, the medium was discarded and replaced with MTT solution (500 μg/ml) and incubated for 8 hrs. After removing the MTT solution, the crystals were dissolved in DMSO, and the absorbance was measured at 550 nm. The percentage of cell viability was calculated using the following formula: treating/untreated × 100%, and the data were presented as mean ± SD. The experiment was repeated three times under the same conditions.

### Synergistic proliferation assay

Cells (5 × 10^3^) were seeded in 96-well plates and treated with serial concentrations of JIB extract (0-60 μg/ml) and cisplatin (0-4 μg/ml). Cell viability inhibition was determined using the MTT assay, and the combination index was calculated using CompuSyn software (ComboSyn, Inc., Paramus, NJ, USA) 24, 25. Each experiment was carried out in triplicate, independently.

### Cell cycle analysis

B16/F10 cells were seeded in 10 cm dishes at a density of 1 × 10^6^ cells and cultured for 12-16 hrs in an incubator. After cell fulling to 60%-70% of dishes, the indicating drugs were given to cells for 6, 12, 24 and 48 hrs. After treatment, the cells were harvested and stained with PI (40 μg/ml) in PBS containing 100 μg/ml RNase overnight at 4°C. Cell cycle distribution was evaluated in independent tests for three times. The detailed analyzed method we utilized in this experiment was described as the following. We selected a fixed area of sub-G_1_, G_0_/G_1_, S, and G_2_/M phase in each group. All cells were divided into two groups, and one was the living cell population to establish the purpose of observing drug-induced changes of the cell population in cell cycle distribution. The calculated formula of cell cycle distribution was the indicated cell population (G_0_/G_1_, S, or G_2_/M phase) / total alive cells × 100%. Another was a dead cell population to evaluate drug-induced cell death. The area of sub-G_1_ phase was set below 160 of FL2-A and the calculated formula of sub-G_1_ phase was sub-G_1_ cell population / total cell population containing alive and dead cells × 100%. Cell cycle distribution was determined using a FACSCalibur (Franklin Lakes, NJ, USA) and analyzed using FlowJo 7.6.1 (Ashland, Oregon, USA).

### Western blot

B16/F10 cells (1 × 10^6^/dish) were treated with JIB extract (30 μg/ml), cisplatin (2 μg/ml), and a combination of drugs. After treatment, the cells were harvested and incubated with lysis buffer to collect the total protein extract. Total protein concentration was determined using BCA Protein Assay Reagent (Thermo Fisher Scientific, Waltham, MA, USA). Proteins were electrophoretically separated on SDS-PAGE and transferred onto PVDF. The membranes were incubated with 5% skim milk to block nonspecific binding. Primary and secondary antibodies were added to incubate with membranes for the desired temperature and time points, respectively. After washing, the membranes were incubated with horseradish peroxidase and visualized by chemiluminescence (T-Pro LumiFast Plus Chemiluminescence Detection Kit; T-Pro Biotechnology, New Taipei County, Taiwan). Immunolabeled proteins were detected by GE LAS-4000 (Little Chalfont, United Kingdom), and the intensities of stained bands were quantified using the ImageJ software (NIH, Betlesda, MD, USA). Three parallel experiment analyses are carried out. The primary antibodies against ERK, PARP, p21, bax, bcl2, AKT, cyclinB1 and cyclinD1 were purchased from iReal Biotechnology Co., Ltd. (Hsinchu, Taiwan) and the others were purchased from Santa Cruz Biotechnology (California, USA).

### Statistical analysis

Quantitative data were presented as mean ± SD. Results were statistically analyzed using the Student's *t*-test. A *P* value of <0.05 was considered significant.

## Results

### JIB extract reduced the viability of B16/F10 cells and affected the MDCK cell growth with less inhibitory effects

**Figure 1:**
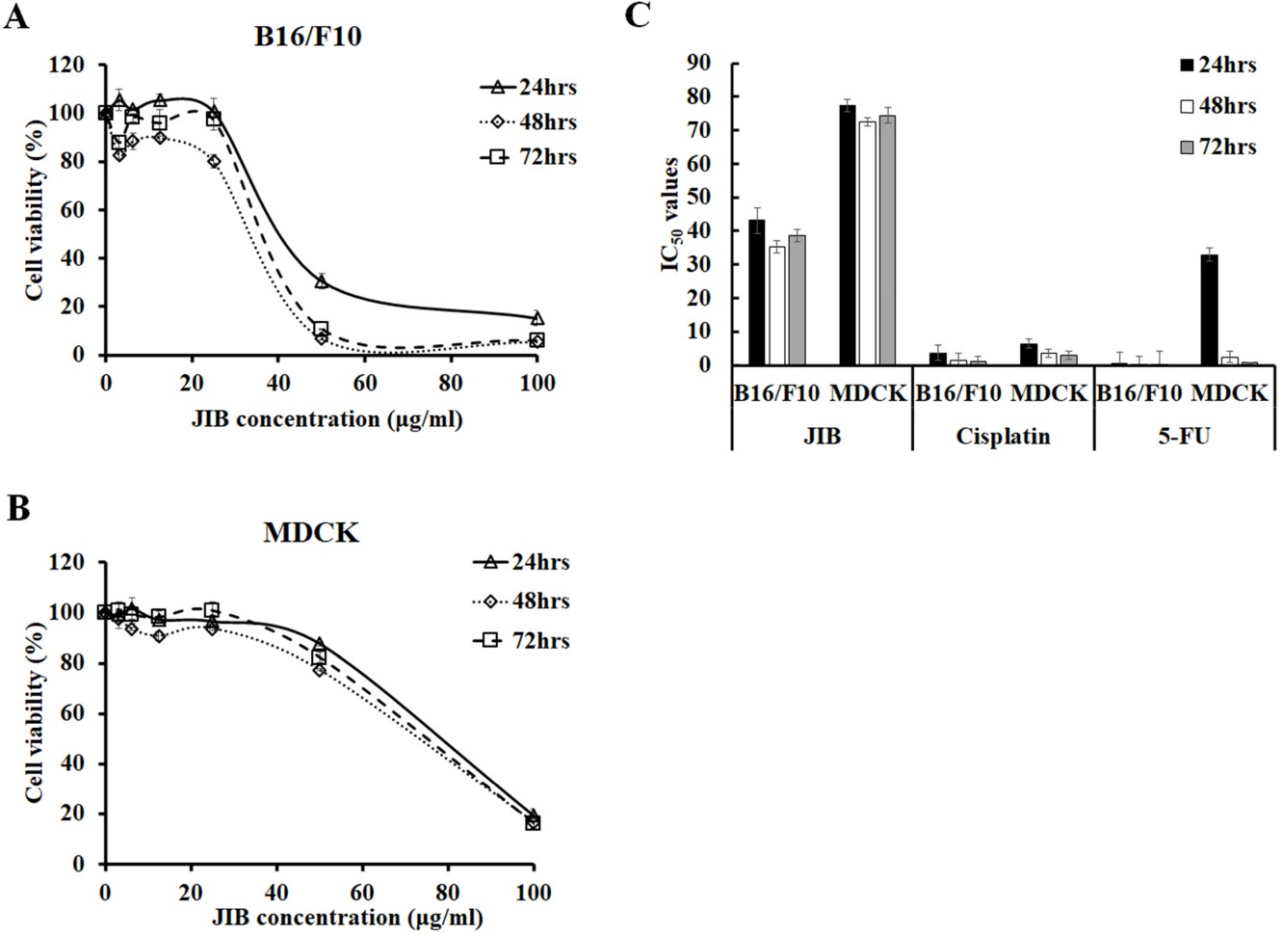
** JIB extract inhibited B16/F10 cells proliferation and had a lower inhibitory effect on MDCK cells.** B16/F10 (5 × 10^3^) and MDCK (1 × 10^4^) cells were seeded in 96-well plates and treated with JIB extract (0-100 µg/ml) for 24, 48, and 72 hrs. Cell viability was determined as a percentage and was evaluated by the MTT assay. (**A,B**) Growth inhibition of B16/F10 and MDCK cells. (**C**) The IC_50_ of B16/F10 and MDCK cells were treated with JIB extract, cisplatin, or 5-FU for 24, 48, and 72 hrs.

To examine the inhibitory effect of JIB extract on B16/F10 cells, B16/F10 cells were treated with serial concentrations of JIB extract for the indicated time, and the MTT assay was performed. The cell viability of B16/F10 cells was reduced from 100% to 15.22 ± 3.28%, 5.43 ± 0.16%, and 5.86 ± 0.25% after treatment with 0-100 μg/ml JIB extract for 24, 48, and 72 hrs, respectively. Moreover, 50 and 100 μg/ml of JIB extracts inhibited the cell viability of B16/F10 cells by 69%-94% in a dose-dependent manner (Figure 1A). The JIB extract-treated MDCK cells were further testified and MDCK cells derived from *Canine* strain were used to evaluate the kidney toxicity *in vitro*. As shown in Figure 1B, 100 μg/ml JIB extract strongly inhibited the cell viability of MDCK cells by 80%. However, 50 μg/ml JIB extract inhibited 12%-22% of the MDCK cells. In addition, the IC_50_ of JIB extract in B16/F10 cells was 43.12 ± 3.81 μg/ml at 24 hrs. Compared with the IC_50_ of JIB extract, the IC_50_ value of MDCK cells was 77.48 ± 1.81 μg/ml at 24 hrs and was approximately 2-fold higher than the IC_50_ of B16/F10 cells (Figure 1C). These results revealed that JIB extract at concentrations of 50 and 100 μg/ml efficiently inhibited B16/F10 cells but not MDCK cells, suggesting that JIB extract might present a good selective ability for tumor cells owing to the significant difference in IC_50_. Subsequently, the IC_50_ values of cisplatin and 5-FU, the chemotherapeutic drugs that are commonly used for combination therapy, were determined by the MTT assay. Although cisplatin and 5-FU showed a better inhibitory effect on B16/F10 cells, these chemotherapeutic drugs also exerted strong cytotoxicity against MDCK cells owing to the IC_50_ of cisplatin and 5-FU, showing no obvious difference in both cell types. In summary, JIB extract inhibited B16/F10 cell growth and was less cytotoxic to MDCK cells.

### JIB extract plus cisplatin showed a synergistic effect to decrease cell viability in B16/F10 cells

**Figure 2:**
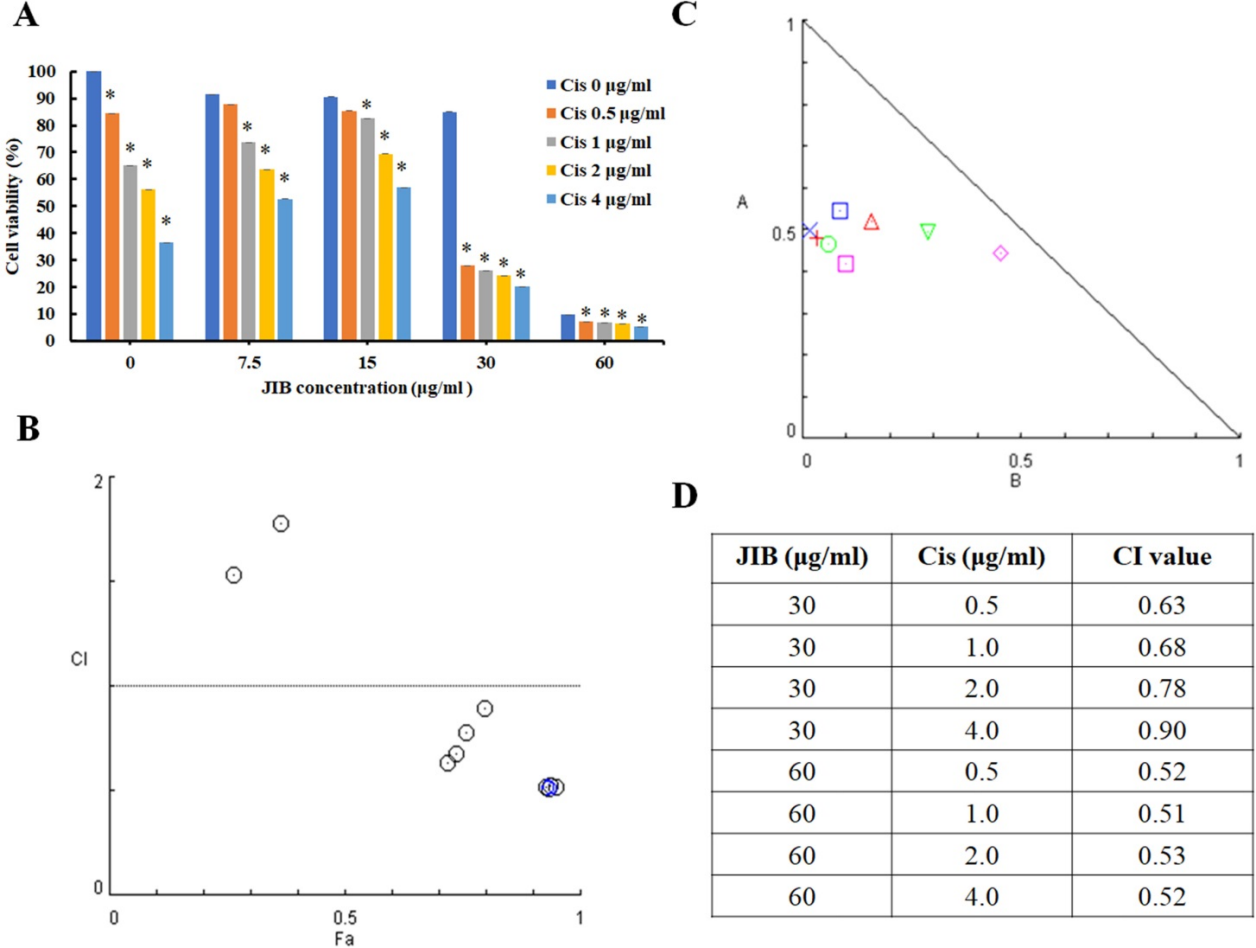
** JIB extract combined with cisplatin had a synergistic effect on the inhibition of B16/F10 cell growth.** B16/F10 cells with a density of 5 × 10^3^ cells were grown in 96-well plates and treated with JIB extract (0, 7.5, 15, 30, and 60 µg/ml) and cisplatin (0, 0.5, 1, 2, and 4 µg/ml) for 48 hrs. The synergistic effect was expressed as a percentage of cell viability and evaluated by the MTT assay. (**A**) The cell viability of JIB extract combined with cisplatin on B16/F10 cells. *: Indicates a significant decrease between untreated and treated groups (*p* < 0.05). (**B**) Combination index plot. (**C**) Normalized isobologram for the combination of JIB extract and cisplatin. (**D**) The table of the combination index.

Cisplatin is utilized as a part of combination therapy to treat melanoma, and the common adverse effects are immunosuppression and nephrotoxicity. The former data revealed the anti-melanoma activity of JIB extract. To evaluate the combination index of JIB extract plus cisplatin, the B16/F10 cells were treated with combination treatment for 48 hrs. As shown in Figure 2A, cisplatin combined with JIB extract of 7.5 and 15 μg/ml did not definitely reduce cell viability. Nevertheless, when cisplatin (0.5-4 μg/ml) was combined with 30 μg/ml of JIB extract, the cell viability of B16/F10 cells decreased ranged from 67% to 76% (Figure 2A). After that, the combination index was calculated using the Comsyn software. The combination index plot and normalized isobologram showed that JIB extract combined with cisplatin had a synergistic effect on the inhibition of B16/F10 cell growth within 48hrs (Figure 2B and C). In addition, JIB extract concentrations of 30 and 60 μg/ml revealed serial combination index, which was less than 1, while JIB extract combined with serial cisplatin (Figure 2D). These results demonstrated that JIB extract plus cisplatin exhibited a synergistic effect.

### JIB extract combined with cisplatin to impede cell cycle progression and downregulate cell cycle-related proteins

**Figure 3:**
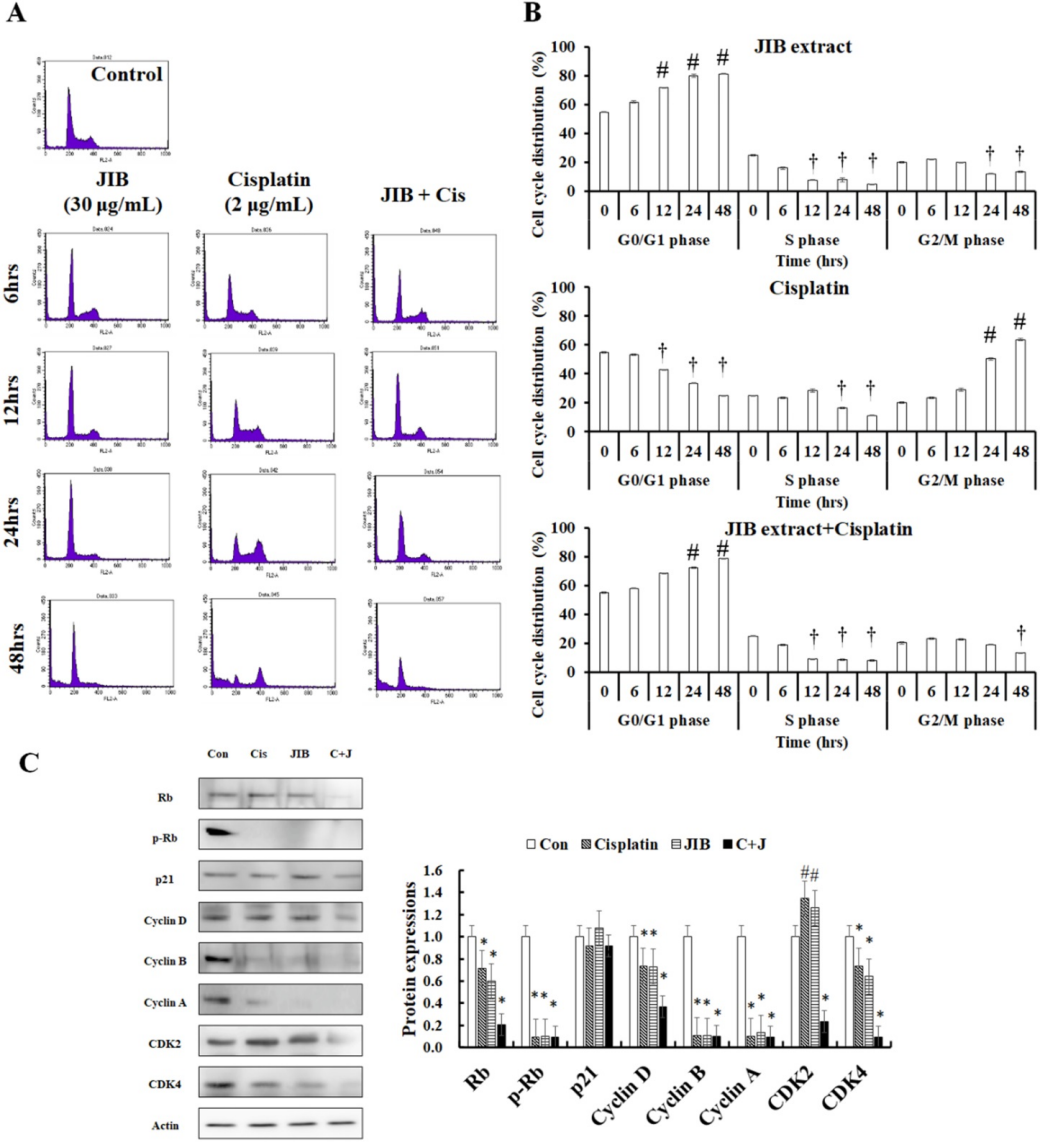
** JIB extract synergized with cisplatin to block cell cycle progression at the G_0_/G_1_ phase in B16/F10 cells.** B16/F10 cells (1 × 10^6^) were seeded in 10 cm dishes and treated with a combination of JIB extract (30 µg/ml) and cisplatin (2 µg/ml) for 6, 12, 24, and 48 hrs. After harvesting cells and staining with PI, the cell cycle progression of B16/F10 cells was analyzed by flow cytometry with the channel at FL2-A (red). (**A**) Cell cycle distribution plot. (**B**) The bar of cell cycle distribution on JIB extract, cisplatin, and a combination of JIB extract and cisplatin. #: It was a significant increase between untreated and treated groups; †: It was a significant decrease between untreated and treated groups (*p* < 0.05). After drug treatments, total proteins were harvested, quantified by the BCA assay, and resolved by Western blotting. (**C**) The expression of cell cycle-related proteins in B16/F10 cells. C+J: indicated cisplatin treatment plus JIB treatment. *: It was a significant increase between untreated and treated groups; #: It was a significant decrease between untreated and treated groups (*p* < 0.05).

Former results indicated that JIB extract combined with cisplatin had a synergistic effect; to examine whether the combination treatment affected the cell cycle distribution of B16/F10 cells, flow cytometry was conducted. JIB extract, cisplatin, and combination treatment affected the cell cycle distribution at different time points (Figure 3A). JIB extract induced cell cycle arrest at the G_0_/G_1_ phase in a time-dependent manner and these values were showed 54.91 ± 0.39%, 61.81 ± 0.76%, 71.94 ± 0.22%, 79.99 ± 1.23%, and 81.37 ± 0.43% corresponding to the indicated time interval and reduced the population in the S phase and G_2_/M phase, respectively. Cisplatin increased the cell population in the G_2_/M phase within at 24 and 48 hrs (20.51 ± 0.69%, 23.51 ± 0.38%, 29.00 ± 1.18%, 50.45 ± 0.79, and 63.69 ± 0.64%) and decreased the population in the G_0_/G_1_ and S phase. JIB extract synergized with cisplatin to increase cell cycle arrest at the G_0_/G_1_ phase (54.91 ± 0.39%, 57.84 ± 0.27%, 68.36 ± 0.23%, 72.40 ± 0.40%, and 78.91 ± 0.07%) and reduced the S and G_2_/M cell populations (Figure 3B). These results suggested that JIB extract plus cisplatin increased the majority of the cell population in the G_0_/G_1_ phase to inhibit B16/F10 cell growth. Next, we tested the cell cycle-related proteins that might be regulated by JIB extract plus cisplatin. Rb binds to the E2F factor to block cell cycle progression and phosphorylated Rb is dissociated with E2F to promote cell proliferation 26. After treatment with JIB extract or cisplatin, JIB extract plus cisplatin inhibited total Rb and phosphorylated Rb expression, thus reducing cell growth. JIB extract strongly induced p21 protein expression, which resulted in cell cycle arrest. The expression of downstream cell cycle proteins that are regulated by p21, including cyclins and CDKs were reduced after JIB extract or cisplatin treatment. JIB extract combined with cisplatin enhanced the suppression of cyclins and CDKs protein expressions, especially Rb, cdk2, cdk4, and cyclin D (Figure 3C). JIB extract or cisplatin possessed an anti-proliferative ability through the mediation of cell cycle-related proteins to arrest cell cycle progression, and JIB extract plus cisplatin improved the obstruction of cell cycle progression in B16/F10 cells.

### JIB extract synergized with cisplatin to inhibit AKT/mTOR signaling

**Figure 4:**
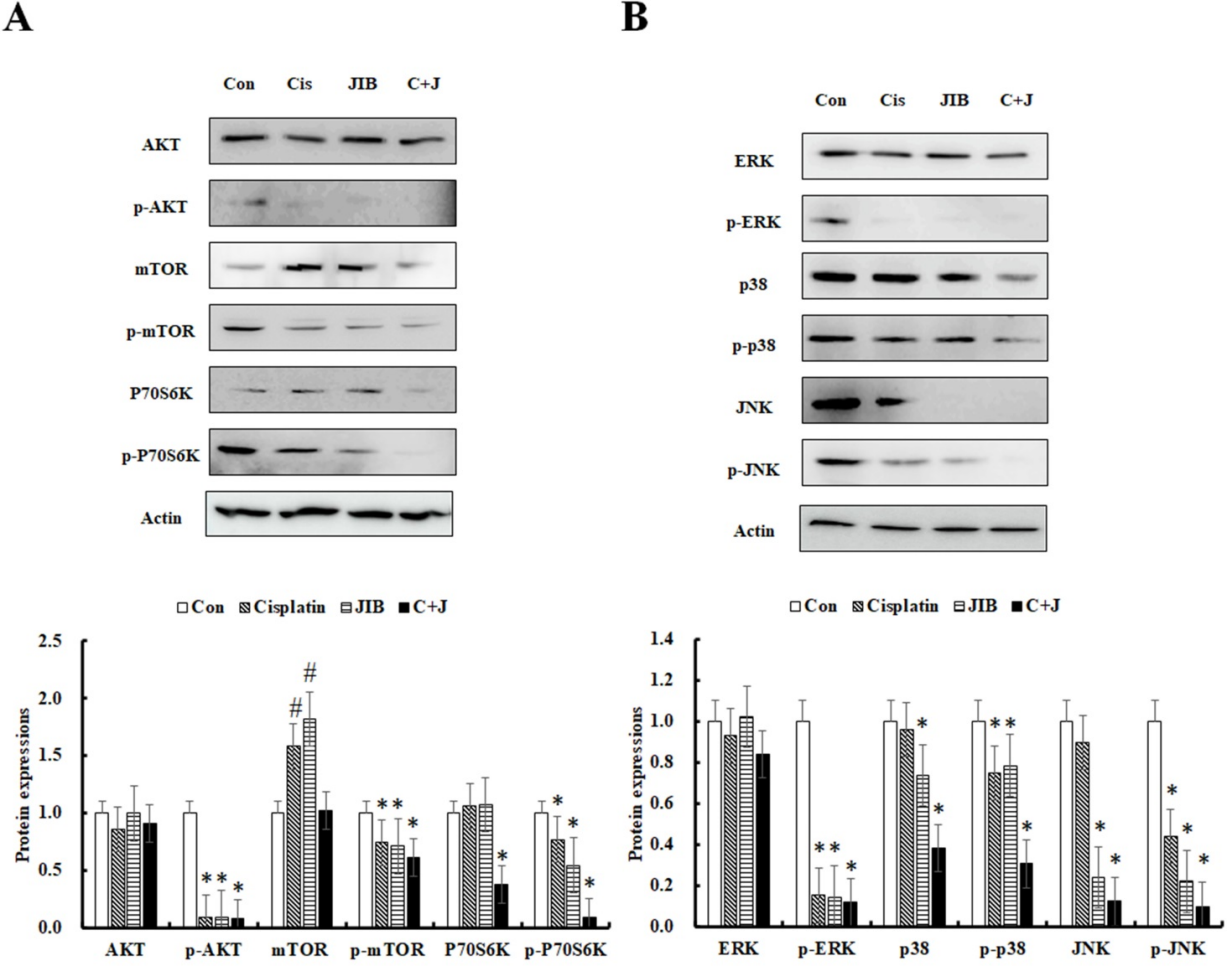
** JIB extract synergized with cisplatin to repress cell proliferation and survival pathways in B16/F10 cells.** B16/F10 cells were treated with the indicated drugs for 48 hrs and harvested to determine the concentration of total proteins. After that, total proteins were analyzed by Western blotting. C+J: indicated cisplatin treatment plus JIB treatment. *: It was a significant decrease between untreated and treated groups; #: It was a significant increase between untreated and treated groups (*p* < 0.05).

The AKT/mTOR pathway is known to facilitate cell proliferation and survival in various cancers. Consequently, the AKT/mTOR pathway was further examined. As shown in Figure 4A, the AKT protein was not clearly reduced by drug alone or a combination of JIB extract and cisplatin. The expression of p-AKT was markedly inhibited by JIB extract, cisplatin and so as in combination treatment. mTOR and p-mTOR (Ser2448) proteins were strongly decreased by treatment with JIB extract plus cisplatin in comparison with drug alone treatment. Similar results were obtained for the downstream proteins of P70S6Kα and p-P70S6Kα (Ser411), which were strongly repressed by combination treatment compared with either drug alone (Figure 4A). The results indicated that JIB extract combined with cisplatin exerted a synergistic effect to impede the AKT/mTOR pathway, thus inhibiting cell proliferation and growth.

### JIB extract synergized with cisplatin to reduce ERK, p38, and JNK signaling

The MAPK pathway, including ERK, p38, and JNK, was further to examine the changes of protein expressions. ERK signaling plays important roles in regulating cell proliferation, and cell survival 27; p38 signaling is often activated in advanced-stage melanoma that correlates to metastatic and chemoresistant melanoma 28; JNK signaling regulates lots of cellular processes, including cell proliferation, differentiation, survival and migration 29. JIB extract or cisplatin only slightly affected ERK and p38 expression. In contrast, JNK expression was distinctly reduced by JIB extract. The phosphorylation of ERK was repressed by both JIB extract and cisplatin; by contrast, p-p38 and p-JNK were not markedly affected by either of the drugs. The combination of JIB extract and cisplatin predominantly decreased the expression of p-ERK1/2 (Tyr204), p-p38α/β/γ (Tyr182), and p-JNK1/2/3 (Thr183/Tyr185) (Figure 4B). Overall, drug alone and combinational treatment significantly interrupted the p-ERK protein expression to suppress B16/F10 cell growth. Besides, p38 signaling was statistical difference inhibited by JIB extract and combinational treatment, suggesting that JIB extract might enhance the anti-melanoma capacity of cisplatin on metastasis and also reduce the potential of chemoresistant in B16/F10 cells. JIB extract alone and its' combinational treatment diminished the JNK signaling in B16/F10 cells to suppress cell proliferation. As a result, the combination of these two drugs strengthened the inhibition of the MAPK pathway.

### JIB extract plus cisplatin promoted apoptosis

**Figure 5:**
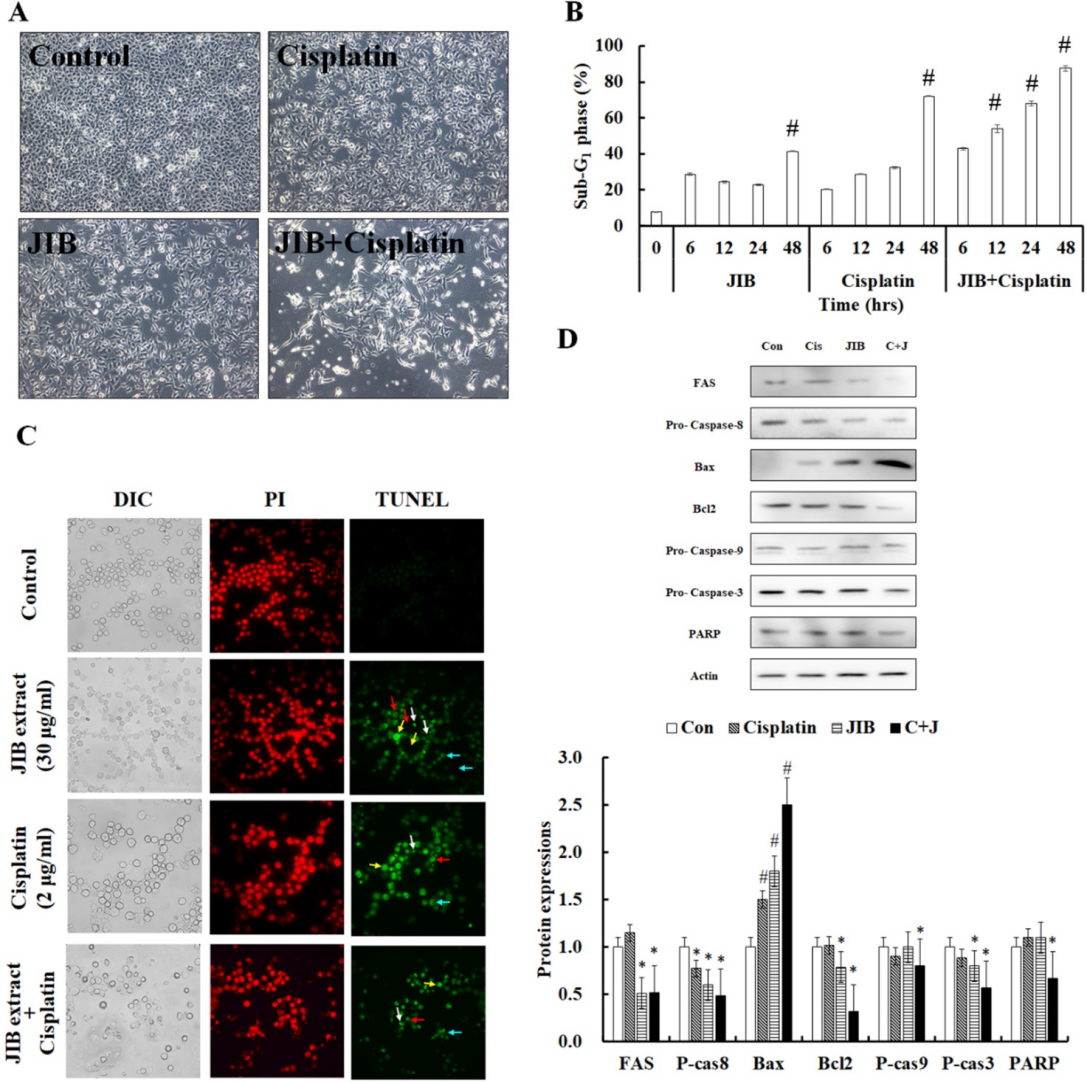
** JIB extract synergized with cisplatin to contribute to cell apoptosis in B16/F10 cells.** B16/F10 cells (1 × 10^6^/dish) were treated with JIB extract, cisplatin, and a combination of JIB extract and cisplatin for 6, 12, 24, and 48 hrs. (**A**) The cell morphology of B16/F10 cells after drug treatment for 48 hrs. B16/F10 cells were treated with drugs and harvested for sub-G_1_ phase analysis by flow cytometry. (**B**) The sub-G_1_ phase of B16/F10 cells after treatment. #: It was a significant increase between untreated and treated groups (*p* < 0.05). (**C**) TUNEL staining of the treatments. Green: TUNEL-positive; red: PI staining as counter staining. White arrow: apoptotic bodies; red arrow: anoikis; yellow arrow: DNA fragments; blue arrow: chromatin condensation. (**D**) The apoptosis-related protein expression in B16/F10 cells treated with drugs for 48 hrs. C+J: indicated cisplatin treatment plus JIB treatment. *: It was a significant decrease between untreated and treated groups; #: It was a significant inecrease between untreated and treated groups (*p* < 0.05).

We examined whether JIB extract plus cisplatin enhanced cytotoxicity in B16/F10 cells. The morphology of B16/F10 cells was examined. As shown in Figure 5A, B16/F10 cells were detached from the dish, cell bodies were elongated, cells were floating and cell debris was generated after combination treatment (Figure 5A). Furthermore, the sub-G1 phase was examined to estimate the ratio of cell death. Results revealed that JIB extract combined with cisplatin unequivocally induced cell death, ranging from 42.76 ± 0.49% to 87.50 ± 1.50%, in a time-dependent manner. JIB extract induced cell death, ranging from 28.60 ± 0.55% to 41.41 ± 0.21%, and cisplatin-induced cell death, ranging from 20.22 ± 0.22% to 72.05 ± 0.30%. Therefore, JIB extract synergized with cisplatin to induce about 1.2- to 2.1-fold of cell death compared with drug-only treatment in B16/F10 cells (Figure 5B). JIB extract combined with cisplatin improved cell apoptosis in B16/F10 cells, and the results showed that JIB extract synergized with cisplatin to promote cell apoptosis (Figure 5C). Hence, the mechanisms of cell apoptosis were further verified, while combination treatment was applied to B16/F10 cells. JIB extract and combination treatment were shown to reduce FAS protein expression. In addition, the expression of procaspase-8 protein was either reduced by JIB extract only or combination treatment, suggesting that the extrinsic pathway might be activated. On the other hand, an increase in bax and decrease in bcl-2 protein expression was induced by JIB extract and combination treatment; by contrast, cisplatin did not show obvious changes in bax and bcl-2 protein expression. The ratio of bax/bcl-2 was increased, and the level of procaspase-9 was reduced, indicating that the intrinsic pathway might be turned on after JIB extract and combination treatment. The upstream apoptotic proteins and caspase were activated, resulting in downstream procaspase-3 and PARP reduction after JIB extract plus cisplatin treatment (Figure 5D). Therefore, JIB extract not only induced cell apoptosis but also synergized with cisplatin to promote cell apoptosis in B16/F10 cells.

## Discussion

The bio-functions of JIB extract remain unclear on anti-cancer capacity; as a result, in this study, we first demonstrated the anti-melanoma activity of JIB extract. Current therapy for melanoma usually causes several side effects. For example, vemurafenib triggers the development of hand and foot syndrome 30-32, cisplatin causes nephrotoxicity and immunosuppression 33. Additionally, drug resistance might lead to ineffective treatment. As a result, taking the abovementioned problems into consideration, JIB extract induced more cytotoxicity of B16/F10 cells than MDCK cells. This result suggested that the toxicity of JIB extract was mitigated the injury of MDCK cells. However, we utilized only B16/F10 cells derived from mouse strain and MDCK cells were cell lines derived from *Canine* strain and the more types of cell lines would need to verify the cytotoxicity of JIB extract for the future experiment.

Single drug treatment commonly impedes one or a few specific signaling pathways, which increases the risk of drug resistance. As a result, recently, combination therapy is preferred as a therapeutic strategy due to the inhibition of multiple signaling pathways preventing drug resistance, and drug dosage is reduced to alleviate unpredictable side effects 34. Our results indicated that JIB extract exhibited its synergistic effects with cisplatin. Furthermore, JIB extract (30 μg/ml) combined with cisplatin (0.5 and 1 μg/ml) exhibited a synergistic effect in 48 hrs, and the CI values were 0.63 and 0.68, respectively. The results indicated that the combinational treatment would decrease cisplatin's dose from 2 μg/ml to 0.5 and 1 μg/ml and indirectly reduce the cisplatin-induced toxicity. Besides, the cytotoxicity of JIB to MDCK cells that were used as an example for kidney toxicity assay in this study was lower than cisplatin, presuming JIB extract might be less toxic to normal cells. However, there are not many studies working on the *J. indica* Bertol., and we will further study the new bio-functions of *J. indica* Bertol. in the future. As a result, we provide a linkage possibility of utilizing natural extract in clinical therapy or reduce side effects by combining the JIB extract and cisplatin in melanoma treatment.

The combination treatment of JIB extract and cisplatin enhanced the suppression of cell proliferation by blocking the cell cycle progression, repressing cell growth, and inhibiting cell survival via the AKT/mTOR and MAPK pathway, as well as inducing cell apoptosis through caspase activation. During the development of melanomas, the AKT/mTOR and MAPK pathways promote cell growth, proliferation, migration, apoptosis and survival, and these signaling cascades can promote melanoma metastasis results in poor prognosis 35, 36. AKT is active during mitosis to induce mTOR/P70S6K signaling that promotes protein synthesis and cell growth 37 and can inhibit p21 expression to trigger the formation of an active cyclin D-CDK4/6 complex to proceed cell cycle progression 38. Besides, ERK activation plays a critical role in several levels of cell proliferation by inhibition of CDKIs, an increment of the activity of CDKs, facilitating of cyclin D1 expression and regulation of cyclinE/CDK2 formation to enforce Rb phosphorylation that contributes to G_1_/S transition 27. Literature has revealed previously that ERK and AKT activation have cooperation in inducing cyclin D1 expression. In this study, the drug alone downregulated the phosphorylated AKT/mTOR/P70S6K and ERK protein expressions. The combinational treatment synergistically reinforced the inhibition of activated AKT, mTOR, P70S6K and ERK, strengthened the reduction of phosphorylation of Rb, the levels of cyclins A, B, D, cdk2 and cdk4 and, ultimately, enhanced the obstruction of cell cycle procced to repress cell proliferation and growth in B16/F10 cells.

Subsequently, our results revealed that JIB extract and combinational treatment both induced cell cycle arrest at G_0_/G_1_ phase and cisplatin administration caused G_2_/M phase in B16/F10 cells. The Rb protein is a tumor suppressor and its' phosphorylation can proceed irreversible G_1_/S transition that triggers cell proliferation 26. Drug alone or combinational treatment inhibited the phosphorylation of Rb to reduce cell proliferation in B16/F10 cells. After JIB extract treatment, the level of p21 increased and downstream cyclin and cdk decreased, suggesting JIB extract could affect p21 expression to further regulate the cell cycle progression. However, JIB extract combined with cisplatin not significantly induced p21 expression. In the results, we assumed that the combination of JIB extract and cisplatin was significantly decreased the level of cyclin D, cdk2 and cdk4 contributing to G_0_/G_1_ phase arrest but not p21 expression. Or, JIB extract combined with cisplatin might exert other pathways on the mediation of cell cycle progression that deserved further investigating the detailed mechanisms in future work.

MAPK signaling plays vital roles in the regulation of cell growth, cell survival, cell proliferation, oxidant stress, and ER stress 39. Previous results showed that the MAPK pathway in melanoma cells is highly activated and ERK, p38, and JNK proteins are overexpressed in the tumor mass of patients that are correlated with drug resistance and reduce the survival benefits of patients 40. For instance, BRAF inhibition results in a rapid recovery of phospho-ERK (pERK) signaling result in drug resistance 41; activation of JNK is associated with cell proliferation and shorter relapse-free period for patients with superficial spreading melanomas 42, 43; p38 activity contribute to metastasis result in poor prognosis 44. Numerous observations indicate a growth-promoting role of JNK in various cell types such as hepatocarcinoma cells, fibroblasts, immortalized neural stem cells and melanoma cells and have reported that active ERK indirectly facilitates the JNK activity and enforces JNK-Jun signaling to elevate cyclin D1 expression result in cell cycle progression 45. Additionally, ERK activation is responsible for increasing the interaction between αVβ3 and vitronectin and stimulates p38 activity to increase melanoma cell proliferation 46, 47. Our findings revealed that JIB extract effectively inhibited both p38, JNK and phosphorylated proteins. Furthermore, JIB extract combined with cisplatin not only significantly strengthened to reduce upstream of activated AKT and ERK proteins but also to repress downstream of activated p38 and JNK proteins. Consequently, we suggested that JIB extract potentiated cisplatin-mediated cytotoxicity through enhanced growth inhibition and increased apoptotic induction. In subsequent results, JIB extract effectively reduced the level of procaspase-8, suggesting JIB extract might activate the extrinsic apoptotic pathway. On the contrary, JIB extract increased bax/bcl2 ratio, suggesting that JIB extract might lightly induce intrinsic apoptotic apoptosis. JIB extract finally activated the caspase cascade lead to cell apoptosis. After that, JIB extract combined with cisplatin dramatically enhanced the apoptosis of B16/F10 cells through the increment of the extrinsic and intrinsic apoptotic pathways.

In conclusion, JIB extract combined with cisplatin had synergistic effects on the suppression of tumor cell growth and induction of apoptosis in B16/F10 cells through different molecular pathways, including AKT/mTOR, MAPK pathways, cell cycle arrest and caspase-dependent pathways. Consequently, JIB extract can be potentially used as an anti-tumor drug and adjuvant therapy for melanoma.
